# Identity profiles and wellbeing of Italian student-athletes

**DOI:** 10.3389/fspor.2026.1817619

**Published:** 2026-06-29

**Authors:** Giampaolo Santi, Roberto Roklicer, Ross Wadey, Attilio Carraro

**Affiliations:** 1 Faculty of Education, Free University of Bolzano-Bozen, Brixen, Italy; 2 Department of Sport and Exercise Sciences, St. Mary’s University, London, United Kingdom

**Keywords:** dual career, mental health, performance, psychological wellbeing, social wellbeing, subjective wellbeing

## Abstract

**Introduction:**

Student-athletes balance academic and athletic commitments, thus developing different combinations of academic and athletic identity. The present study investigates these different identities and examines them in relation to satisfaction with academic and sport performance and well-being.

**Methods:**

A total of 223 Italian student-athletes completed a set of self-report measures: the Academic and Athletic Identity Scale (AAIS), two single items measuring satisfaction with academic and sport performance, the Athlete Psychological Well-Being Inventory (APWBI), and the Mental Health Continuum–Short Form (MHC-SF).

**Results:**

Four identity profiles were identified: “fully engaged student-athletes” (*n* = 79), “committed athletes” (*n* = 28), “academics-focused student-athletes” (*n* = 39), and “academically unengaged athletes” (*n* = 77). These four groups differed in terms of satisfaction with academic (*p* < .001) and sport (*p* < .001) performances, precursors of well-being, athletic well-being (*p* < .001), and mental health (*p* = .001), with disengagement being generally indicative of decreased satisfaction and poor well-being in one or more areas.

**Conclusion:**

In conclusion, the present study highlights how different identity profiles correspond to diverse conditions of wellbeing and may suggest directions for intervention.

## Introduction

Student-athletes in the university age group face high demands due to their dual commitments in sport and education. In reality, student-athletes encounter additional challenges beyond those faced by their peers, including increased risk of injury, complex time management demands, and identity-related issues (1, 2). Research in sport and education has highlighted how social identities, in particular, are protective factors against some of these stressors (3, 4). Social identity, as proposed by Tajfel and Turner (5, 6), refers to the identity that individuals develop by being or feeling part of a group, such as racial/ethnic and gender identities.

The scientific literature indicates that a cohesive and integrative sense of self or identity is positively associated with an individual's wellbeing (7–9). For example, Meeus et al. (7) explored work identity in association with psychological wellbeing among young Dutch adults. Their results highlighted a strong work identity was linked with high levels of psychological wellbeing. Similarly, Sharma and Chandiramani (8) reported a positive association between ego identity and psychological wellbeing in Indian adolescents. Ward and Szabó (9), in their review, explored the association between wellbeing and cultural identity, a type of social identity that encompasses ethnic, national, and religious identities. The authors stated that a coherent sense of self—that is, a strong and integrated identity—is generally associated with greater wellbeing.

Limited research, however, has focused on the identity of student-athletes in relation to their wellbeing (2, 10). Miller and Hoffman (10), in their study on college students, emphasised that athlete identity was related to lower risk of depression and reduced thoughts of suicide. Parker et al. (2), on the other hand, focused on the social identity among student-athletes and found that it may help reduce stress during important educational transitions (i.e., from high school to the university).

An additional issue pertains to the operationalisation of student-athlete identity. Parker et al. (2), for example, adapted a single-item measure of social identification as a student-athlete (11). In spite of the validity of this approach, it raises concerns about whether a single item can capture multiple and diverse identities. More recently, Yukhymenko-Lescroart (12) developed the Academic and Athletic Identity Scale (AAIS; 13) to define different identity profiles of student-athletes. In particular, she identified four clusters, labelled “fully engaged college athletes” with high academic and athletic identities; “committed athletes” characterised by low academic identity and high athletic identity; “academics-focused college athletes” with high academic identity and low athletic identity; and “academically unengaged athletes” with low academic and athletic identities (12). In the present study, this categorisation has been adopted to classify Italian student-athletes.

### Aims and hypotheses

The present study aims to examine how different identity profiles of Italian student-athletes differ in terms of satisfaction with sport and academic performance (precursors of wellbeing) and overall wellbeing. Hypotheses are aligned with a previous study protocol (14). In particular, the following findings are expected from the study:

(H1) Student-athletes with a high academic identity (i.e., “fully engaged student-athletes” and “academics-focused student-athletes”) are expected to report greater satisfaction with academic performance than the two groups with lower academic identity (“committed athletes” and “academically unengaged athletes”).

(H2) Student-athletes with a high athletic identity (i.e., “fully engaged student-athletes” and “committed athletes”) are expected to report greater satisfaction with sport performance than the two groups with lower athletic identity (“academics-focused student-athletes” and “academically unengaged athletes”).

(H3) “Fully engaged student-athletes” are also expected to report greater wellbeing than the other three groups of student-athletes.

## Method

### Sample size estimation and justification

An *a priori* sample size estimation was performed using G*Power (15) to determine the required sample size for this study. Power analyses, with alpha error probability set at 0.05 and statistical power set at 0.95, indicated that a minimum of 112 participants would be sufficient to detect large effects in one-way analyses of variance (ANOVAs) with four groups. On the other hand, 132 participants would be required to detect large effects in multivariate analysis of variance (MANOVA) with four groups and three output variables. After data cleansing, a sample of 223 student-athletes was retained for statistical analyses. Sensitivity analysis (*p* = 0.05; 1−*β* = 0.95) indicated that even smaller effects (*F* > 2.646 for ANOVAs; *F* > 1.894 for the MANOVA) could be detected with a sample size of 223 participants.

### Participants

A total of 275 student-athletes were recruited for participation in this study. Of these, 81.1% completed all psychometric scales and were retained in the final sample, which consisted of 223 student-athletes (141 men, 79 women, 3 other gender identity) with a mean age of 25.9 years (SD = 4.7) and 6.4 years (SD = 5.4) of experience in competitive sport. Participants trained an average of 11.2 h/week (SD = 8.8) and were competing in various individual and team sports at international (*n* = 12), national (*n* = 27), regional (*n* = 52), or local (*n* = 132) levels. There were 29 first-year students, 54 second-year, 55 third-year, 25 fourth-year, 33 fifth-year, and 3 sixth-year students (which is possible in medical faculties in Italy). In addition, 24 participants were enrolled in university but had exceeded the typical fifth or sixth year study duration.

### Measures

#### The Academic and Athletic Identity Scale

The AAIS (13) was adapted into the Italian language and adopted for measuring student-athlete identity. The scale begins with the question “How central to your sense of who you are is each of the following attributes?” and comprises 11 items rated on a 6-point Likert scale ranging from 1 to 6. Of these, five items assess academic identity (e.g., “being a capable student,” “doing well in school”), while the remaining six evaluate athletic identity (e.g., *“being a capable athlete,” “being athletic”*). Yukhymenko-Lescroart reported a good fit to the data [*χ*^2^(43, *N* = 596) = 93.56, *p* *<* 0.001, comparative fit index (CFI) = .976, Tucker-Lewis index (TLI) = 0.969, root mean square error of approximation (RMSEA) = 0.044, 90% CI (0.032–0.057), standardised root mean square residual (SRMSR) = 0.026], and high reliability for both subscales, with omega values of 0.93.

#### Satisfaction with academic performance

Satisfaction with academic performance was measured using a visual analogue scale (VAS) ranging from 0 (*Totally dissatisfied*) to 10 (*Totally satisfied*). Participants responded to the question “During my studies at high school or university over the past month, my academic performance left me feeling*…*”. This VAS was adapted from the scale of Arnold et al. (16) on satisfaction with sport performance.

#### Satisfaction with athletic performance

Satisfaction with athletic performance was measured through the VAS of Arnold et al. (16), with responses ranging from 0 (*Totally dissatisfied*) to 10 (*Totally satisfied*). Participants responded to the question “During my sport training and events over the past month, my sporting performance left me feeling*…*”. Subjective measures of performance were adopted instead of objective assessments to facilitate comparison among athletes (16).

#### The Athlete Psychological Well-Being Inventory

The Athlete Psychological Well-Being Inventory (APWBI; 17) was translated into Italian and used as a measure of psychological wellbeing among student-athletes. This measure consists of a 24-item scale rated on a 7-point Likert scale (1–7). Examples of items include “I have the ability to manage my emotions in sport” and “I have a good relationship with other people in sport*.*” Validation studies indicated acceptable model fit [*χ*^2^ = 861.139 (476), *χ*^2^/df = 1.809; CFI = 0.91, incremental fit index (IFI) = 0.92; parsimony comparative fit index (PCFI) = 0.785; RMSEA = 0.050 (90% CI = 0.044–0.055); akaike information criterion (AIC) = 1,109.14] and high reliability for the total score (*ω* = 0.91; *α* = 0.91).

#### The Mental Health Continuum—Short Form

A general measure of wellbeing was also employed using the Mental Health Continuum—Short Form (MHC-SF; 18), administered in its validated Italian version (19). This 14-item scale is introduced by the stem “During the past month, how often did you feel*…*” and measures three dimensions of wellbeing: emotional or subjective wellbeing (three items, e.g., “*…* happy”), social wellbeing (five items, e.g., “*…* that you had something important to contribute to society”), and psychological wellbeing (six items, e.g., “*…* that you liked the most parts of your personality”). Answers were given on a 6-point Likert scale (0–5). This scale received strong supporting evidence for validity and reliability: The three-factor structure has been confirmed in various population samples (20, 21), and internal consistency reliability values have been reported as high (>0.80).

### Procedure

The study was conducted in accordance with the Declaration of Helsinki, and ethical approval was obtained from the Ethics Committee of the first author's institution (approval code: UNIBZ_EDU_ ENHANCE_2023_30). Participants who completed the questionnaire package were contacted via social networks or e-mail using a snowball sampling technique. The authors contacted potential participants within their own networks and asked them to promote the survey to other potential participants. In addition, the first author contacted sporting clubs through their social webpages and asked them to send the questionnaire to potential participants, involving more people indirectly.

Inclusion and exclusion criteria were based on a previous study protocol (14). All student-athletes had to (1) be currently enrolled in a university, (2) be registered with a national sport federation, and (3) be at least 18 years of age. Upon accessing the survey package, participants read the informed consent form. Only those who provided consent were allowed to complete the survey. Participants were informed that they could withdraw from the survey at any time should they experience psychological discomfort or stress.

### Data analysis

The dataset was first analysed using IBM SPSS 29.0 to explore data distribution and reliability of the scales. The factor structure of the translated scales was then tested through Confirmatory Factor Analysis (CFA) using IBM AMOS Graphics 29.0. Data were then transferred again to IBM SPSS 29.0, and participants were grouped according to their identity profiles. Finally, three ANOVAs and one MANOVA were run to observe differences between the identity profiles group in terms of satisfaction with sport performance, satisfaction with academic performance, athlete psychological wellbeing, and the three dimensions of the mental health continuum. The alpha level for statistical significance was set at *p* < 0.05.

## Results

All items were normally distributed, with skewness and kurtosis values falling within the range of ±2.00 (22). CFAs were conducted on the translated scales to test their structural validity in the Italian sample. CFA for the AAIS indicated good factor structure [model fit: *χ*^2^ = 78.423(41), *χ*^2^/df = 1.913; CFI = 0.98, IFI = 0.98; PCFI = 0.73; RMSEA = .060 (90% CI = 0.040–0.080); AIC = 150.42] (22), with factor loadings ranged from 0.64 to 0.91. The two subscales had good reliability for the academic identity (*ω* = 0.89; *α* = 0.90) and the athletic identity (*ω* = 0.93; *α* = 0.93) (23). CFA for APWBI indicated an acceptable factor structure [model fit: *χ*^2^ = 522.875(235), *χ*^2^/df = 2.225; CFI = 0.92, IFI = 0.92; PCFI = 0.72; RMSEA = 0.070 (90% CI = 0.062–0.078); AIC = 700.88], with all factor loadings ranged from 0.59 to 0.86. Internal consistency reliability for the total score was high (*ω* = 0.95; *α* = 0.95).

Participants were then categorised into high and low academic identity groups based on the median score (4.40) of the AAIS academic identity subscale. Similarly, they were divided into high and low academic identity groups based on the median for (4.17) of the athletic identity subscale. Following Yukhymenko-Lescroart’s categorisation, four identity profiles were identified (12): “fully engaged student-athlete” (*n* = 79) with academic identity and athletic identity above the median, “committed athlete” (*n* = 28) with athletic identity above the median and academic identity below the median, “academics-focused student-athlete” (*n* = 39) with academic identity above the median and athletic identity below the median, and “academically unengaged athletes” (*n* = 77) with both student and athletic identity below the median. See Tables 1, 2 for a summary of these groups.

**Table 1:** Groups based on academic and athletic identities.

Group	Academic identity (scale range 1–5)	Athletic identity (scale range 1–5)
Fully engaged student-athletes (*n* = 79)	>4.40	>4.17
Committed athletes (*n* = 28)	<4.40	>4.17
Academics-focused student-athletes (*n* = 39)	>4.40	<4.17
Academically unengaged athletes (*n* = 77)	<4.40	<4.17

**Table 2:** Distribution of competitive levels across identity profiles.

Identity profile	Competitive level				Total
	International	National	Regional	Local	
Fully engaged student-athletes	6	10	21	42	79
Committed athletes	1	7	10	10	28
Academics-focused student-athletes	0	5	6	28	39
Academically unengaged athletes	5	5	15	52	77
Total	12	27	52	132	223

Three ANOVAs and one MANOVA were run to observe differences between the identity profiles group. The first two ANOVAs tested differences in terms of satisfaction with sport and academic performance. Results from the first analysis of variance (ANOVA) indicated significant differences [*F*_(3, 222)_ = 6.68, *p* = 0.0002, *_p_η*^2^ = 0.08, observed power = 0.97] in terms of satisfaction with sport performance. Bonferroni *post hoc* analyses indicated that “fully engaged student-athletes” reported greater satisfaction with sport performance compared with “academics-focused student-athletes” (*p* = 0.005) and “academically unengaged athletes” (*p* = 0.0007), with “committed athletes” scoring between the other profiles but not significantly differing. Similar results emerged from the second ANOVA [*F*_(3, 222)_ = 19.10, *p* < 0.0001, *_p_η*^2^ = 0.21; observed power > 0.99], which highlighted differences in satisfaction with academic performance. Bonferroni *post hoc* analyses indicated that “fully engaged student-athletes” and “academics-focused student-athletes” reported greater satisfaction with academic performance than the other two groups. Full results are reported in Table 3 and in Supplementary File S1.

**Table 3:** Means, standard deviation, and distribution for each variable across identity profiles.

Variable and identity profile	*N*	*M*	SD	Skewness	Kurtosis
Satisfaction with sport performance (0–10)					
Fully engaged student-athletes	79	6.92^a^	1.86	−0.78	0.40
Committed athletes	28	6.46^a,b^	2.10	−1.18	0.81
Academics-focused student-athletes	39	5.54^b^	2.34	−0.76	0.61
Academically unengaged athletes	77	5.61^b^	2.16	−0.93	0.30
Satisfaction with academic performance (0–10)					
Fully engaged student-athletes	79	7.47^a^	1.77	−0.99	1.59
Committed athletes	28	5.36^b^	2.51	−0.65	−0.10
Academics-focused student-athletes	39	7.36^a^	2.24	−1.21	1.24
Academically unengaged athletes	77	5.29^b^	2.17	−0.48	−0.03
Athletic wellbeing (1–7)					
Fully engaged student-athletes	79	5.76^a^	0.78	−0.52	0.18
Committed athletes	28	5.48^a^	0.68	−0.15	0.67
Academics-focused student-athletes	39	5.45^a^	0.74	−0.12	−0.54
Academically unengaged athletes	77	4.68^b^	0.98	0.26	−0.07
MHC-SF: subjective wellbeing (0–5)					
Fully engaged student-athletes	79	3.49^a^	1.03	−1.09	2.20
Committed athletes	28	3.31^a,b^	1.23	−0.80	0.27
Academics-focused student-athletes	39	3.32^a,b^	0.96	−0.62	0.23
Academically unengaged athletes	77	2.89^b^	0.95	−0.05	0.18
MHC-SF: social wellbeing (0–5)					
Fully engaged student-athletes	79	2.63^a^	1.18	−0.17	−0.85
Committed athletes	28	2.41^a^	1.10	0.03	−0.13
Academics-focused student-athletes	39	2.28^a^	1.07	−0.11	−0.58
Academically unengaged athletes	77	2.35^a^	0.88	0.08	0.82
MHC-SF: psychological wellbeing (0–5)					
Fully engaged student-athletes	79	3.41^a^	1.02	−0.70	0.51
Committed athletes	28	3.18^a,b^	0.98	−0.25	0.06
Academics-focused student-athletes	39	3.15^a,b^	0.98	−0.51	−0.17
Academically unengaged athletes	77	2.73^b^	0.89	−0.12	0.65

Theoretical scale ranges for each variable are indicated in parentheses next to the variable name. Groups sharing a common superscript letter are not significantly different based on Bonferroni *post hoc* analyses.

The third ANOVA explored differences between the four groups in terms of athlete psychological wellbeing measured with the APWBI. Results [*F*_(3, 222)_ = 23.03, *p* < 0.0001, *_p_η*^2^ = 0.24; observed power > 0.99] indicated significant differences, and Bonferroni *post hoc* analyses showed that “academically unengaged athletes” reported lower levels of athlete psychological wellbeing compared with the other three groups (see Table 3 and the Supplementary File S1).

A MANOVA was conducted to examine differences between the groups in terms of mental health, i.e., subjective wellbeing, social wellbeing, and psychological wellbeing. Results indicated a significant model [Wilks’ *λ* = 0.883, *F*_(3, 220)_ = 3.07, *p* = 0.001, *_p_η*^2^ = 0.04, observed power = 0.93], with subjects differing in terms of subjective wellbeing [*F*_(3, 222)_ = 4.73, *p* = 0.003, *_p_η*^2^ = 0.06; observed power = 0.89] and psychological wellbeing [*F*_(3, 222)_ = 6.17, *p* =0 .0003, *_p_η*^2^ = 0.08; observed power = 0.97]. No significant differences emerged for social wellbeing [*F*_(3, 222)_ = 1.32, *p* = 0.269, *_p_η*^2^ = 0.02; observed power = 0.35]. Bonferroni *post hoc* analyses indicated that “fully engaged student-athletes” significantly differed from “academically disengaged athletes” for both subjective and psychological wellbeing. Full results are reported in Table 3 and in Supplementary File S1.

## Discussion

Findings from the present study support the hypotheses that a strong identity is related to increased satisfaction with performance, a precursor of wellbeing. In detail, “fully engaged student-athletes” and “academics-focused student-athletes” reported greater satisfaction with academic performance (H1). Student-athletes with a high athletic identity (i.e., “fully engaged student-athletes” and “committed athletes”) reported greater satisfaction with sport performance (H2). Finally, our hypothesis 3 (H3) was only partially supported. In fact, “fully engaged student-athletes” reported the highest wellbeing scores; however, *post hoc* analyses indicated that differences were significant only in comparison with “academically unengaged athletes,” but not with the other two groups (“academics-focused student-athletes” and “committed athletes”).

In the study by Yukhymenko-Lescroart (12), the four identity profiles were identified through cluster analysis on scalar variables, whereas the present investigation recreated the same profiles based on median splits. Furthermore, Yukhymenko-Lescroart (12) compared these four identity profiles in terms of satisfaction with academia and sport, performance in academia and sport, achievement motivation, and ethical conduct. Conversely, the present study examined differences in satisfaction with academic and athletic performance, as well as wellbeing. A similarity emerged regarding academic performance; specifically, in both studies, “fully engaged student-athletes” and “academics-focused student-athletes” reported the highest scores. It should be noted, however, that Yukhymenko-Lescroart (12) measured performance via self-reported grade point average (GPA), while the present study assessed satisfaction with academic performance. In contrast, results for athletic performance differed: “Academics-focused student-athletes” and “academically unengaged athletes” showed the best individual or team performance in Yukhymenko-Lescroart's (12) study, whereas “fully engaged student-athletes” and “committed athletes” reported greater satisfaction with sport performance in the present investigation. This discrepancy should be interpreted with caution due to the different outcome measures employed.

The present study aimed to compare different student-athletes identity profiles. This approach led to the identification of four distinct identity profiles characterised by varying levels of satisfaction with performance and wellbeing. Consequently, these findings support the notion that student-athlete identity is more than a single social identity, as previously suggested by Parker et al. (2). These authors, in fact, highlighted a linear correlation between the strength of the student-athlete social identity and other psychological outcomes, such as reduced perceived stress. In contrast, the present study conceptualised student identity and athletic identity as distinct yet interrelated constructs, reflecting individuals’ affiliation with two separate social groups. This aligns closely with Tajfel and Turner’s social identity theory (5, 6) and demonstrates that the highest levels of wellbeing are associated with individuals who strongly identify both as students and as athletes.

This study's findings are also in line with the wider scientific literature, which has highlighted strong social identities as protective factors in sport and education (2–4, 10), in work contexts (7), during adolescence (8), and in cultural studies (9). The present research goes beyond these studies by filling a gap in the scientific literature. In particular, it explores the association between distinct student-athlete identity profiles and levels of wellbeing, an area that has received limited attention to date.

### Limitations and future directions

The present study has limitations that should be acknowledged. First, the study did not explore athletic identity foreclosure (24), a dimension of athletic identity that often corresponds to maladaptive adjustments to stressful situations (e.g., sport injury; 25) and may also be related to negative adjustment to dual commitments in sport and education. Future studies, should explore this aspect in depth, possibly including scales that assess foreclosure, such as the Athletic Identity Measurement Scale—third generation (AIMS-3G; 26).

Second, the use of median splits to define groups was adopted to replicate Yukhymenko-Lescroart's (12) categorisation. On the one hand, this choice was made to facilitate comparison with the previous study. On the other hand, it should be acknowledged that our methodology might have reduced variability and affected the results. In addition, the distribution of the sample across four groups resulted in some groups containing a relatively small number of participants.

Finally, the study's findings are based on a heterogeneous sample of student-athletes competing across various sporting levels, from local to international. This inclusive sampling strategy was necessary to achieve an adequate sample size for robust quantitative analyses. However, institutional student-athlete status within Italian universities is typically restricted to those competing at national or international levels (27), who consequently face higher weekly athletic time commitments. Including student-athletes from lower competitive levels may have affected the generalisability of our results. Future research should replicate these findings exclusively within elite cohorts (i.e., national and international levels) who train for a high number of weekly hours. To recruit larger samples of high-level student-athletes, future studies should establish collaborative networks among universities with active dual career programmes.

### Implications for practice

A salient aspect that emerges from the present study is that encouraging student-athletes to excel in both sport and education is beneficial for their wellbeing. Based on this finding, practitioners working with student-athletes (such as sport psychologists, sport scientists, professionals in university counselling services) may be encouraged to support student-athletes in achieving optimal performance in both domains.

## Conclusions

In conclusion, the present study provides valuable insights into the relationships between student-athletes’ identity profiles and their wellbeing. The findings suggest that a pronounced identity in a specific domain tends to align with higher performance satisfaction within that same domain. Furthermore, a more balanced profile—characterised by strong commitments to both academic and athletic roles—appears to be associated with optimal levels of wellbeing. Recognising the methodological boundaries of this research, these preliminary trends suggest that sports psychologists, sports scientists, and academic counsellors might consider encouraging student-athletes to cultivate a dual commitment towards both sport and education.

## Data Availability

The raw data supporting the conclusions of this article will be made available by the authors without undue reservation.
