# Supplementary material for: Identity profiles and wellbeing of Italian student-athletes
Source: Front Sports Act Living. 2026 Jun 29;8:1817619. doi: 10.3389/fspor.2026.1817619 (PMC13359496; doi:10.3389/fspor.2026.1817619)
Supplement: Supplementary file 1 [file Datasheet1.pdf]

Supplementary file – S1

Satisfaction with sport performance

	(I) Group	(J) Group	Mean Difference (I-J)	Std. Error	Sig.	95% Confidence Interval	
						Lower Bound	Upper Bound
Bonferroni	fully engaged student-athletes	committed athletes	.46	.458	1.000	-.76	1.68
		academics-focused student-athletes	1.39*	.408	.005	.30	2.47
		academically unengaged athletes	1.31*	.334	.001	.43	2.20
	committed athletes	fully engaged student-athletes	-.46	.458	1.000	-1.68	.76
		academics-focused student-athletes	.93	.516	.445	-.45	2.30
		academically unengaged athletes	.85	.460	.388	-.37	2.08
	academics-focused student-athletes	fully engaged student-athletes	-1.39*	.408	.005	-2.47	-.30
		committed athletes	-.93	.516	.445	-2.30	.45
		academically unengaged athletes	-.07	.409	1.000	-1.16	1.02
	academically unengaged athletes	fully engaged student-athletes	-1.31*	.334	.001	-2.20	-.43
		committed athletes	-.85	.460	.388	-2.08	.37
		academics-focused student-athletes	.07	.409	1.000	-1.02	1.16

Based on observed means.
The error term is Mean Square(Error) = 4,340.
*. The mean difference is significant at the .05 level.

Satisfaction with academic performance

	(I) Group	(J) Group	Mean Difference (I-J)	Std. Error	Sig.	95% Confidence Interval	
						Lower Bound	Upper Bound
Bonferroni	fully engaged student-athletes	committed athletes	2.11*	.461	.000	.88	3.34
		academics-focused student-athletes	.11	.410	1.000	-.98	1.20
		academically unengaged athletes	2.18*	.336	.000	1.29	3.08
	committed athletes	fully engaged student-athletes	-2.11*	.461	.000	-3.34	-.88
		academics-focused student-athletes	-2.00*	.519	.001	-3.38	-.62
		academically unengaged athletes	.07	.463	1.000	-1.16	1.30
	academics-focused student-athletes	fully engaged student-athletes	-.11	.410	1.000	-1.20	.98
		committed athletes	2.00*	.519	.001	.62	3.38
		academically unengaged athletes	2.07*	.412	.000	.98	3.17
	academically unengaged athletes	fully engaged student-athletes	-2.18*	.336	.000	-3.08	-1.29
		committed athletes	-.07	.463	1.000	-1.30	1.16
		academics-focused student-athletes	-2.07*	.412	.000	-3.17	-.98

Based on observed means.

The error term is Mean Square(Error) = 4,396.

*. The mean difference is significant at the .05 level.

Athlete Psychological Well-Being

	(I) Group	(J) Group	Mean Difference (I-J)	Std. Error	Sig.	95% Confidence Interval	
						Lower Bound	Upper Bound
Bonferroni	fully engaged student-athletes	committed athletes	.2834	.18457	.757	-.2080	.7748
		academics-focused student-athletes	.3070	.16423	.377	-.1302	.7443
		academically unengaged athletes	1.0858*	.13439	.000	.7280	1.4436
	committed athletes	fully engaged student-athletes	-.2834	.18457	.757	-.7748	.2080
		academics-focused student-athletes	.0236	.20787	1.000	-.5298	.5770
		academically unengaged athletes	.8024*	.18519	.000	.3093	1.2954
	academics-focused student-athletes	fully engaged student-athletes	-.3070	.16423	.377	-.7443	.1302
		committed athletes	-.0236	.20787	1.000	-.5770	.5298
		academically unengaged athletes	.7787*	.16493	.000	.3396	1.2179
	academically unengaged athletes	fully engaged student-athletes	-1.0858*	.13439	.000	-1.4436	-.7280
		committed athletes	-.8024*	.18519	.000	-1.2954	-.3093
		academics-focused student-athletes	-.7787*	.16493	.000	-1.2179	-.3396

Based on observed means.
The error term is Mean Square(Error) = ,704.
*. The mean difference is significant at the .05 level.

Mental Health Continuum – Short Form

Dependent Variable	(I) Group	(J) Group	Mean Difference (I-J)	Std. Error	Sig.	95% Confidence Interval	
						Lower Bound	Upper Bound
subjective_ well_being_ Bonferroni MHC_SF	fully engaged student-athletes	committed athletes	.1841	.22471	1.000	-.4141	.7824
		academics-focused student-athletes	.1689	.19995	1.000	-.3635	.7012
		academically unengaged athletes	.6019*	.16362	.002	.1663	1.0375
	committed athletes	fully engaged student-athletes	-.1841	.22471	1.000	-.7824	.4141
		academics-focused student-athletes	-.0153	.25307	1.000	-.6891	.6585
		academically unengaged athletes	.4177	.22547	.392	-.1826	1.0181
	academics-focused student-athletes	fully engaged student-athletes	-.1689	.19995	1.000	-.7012	.3635
		committed athletes	.0153	.25307	1.000	-.6585	.6891
		academically unengaged athletes	.4330	.20080	.193	-.1016	.9676
	academically unengaged athletes	fully engaged student-athletes	-.6019*	.16362	.002	-1.0375	-.1663
		committed athletes	-.4177	.22547	.392	-1.0181	.1826
		academics-focused student-athletes	-.4330	.20080	.193	-.9676	.1016

social_well_being_MHC_SF	Bonferroni	fully engaged student-athletes	committed athletes	.2136	.23231	1.000	-.4050	.8321
			academics-focused student-athletes	.3509	.20671	.546	-.1994	.9013
			academically unengaged athletes	.2746	.16915	.636	-.1758	.7250
		committed athletes	fully engaged student-athletes	-.2136	.23231	1.000	-.8321	.4050
			academics-focused student-athletes	.1374	.26164	1.000	-.5592	.8339
			academically unengaged athletes	.0610	.23310	1.000	-.5596	.6817
		academics-focused student-athletes	fully engaged student-athletes	-.3509	.20671	.546	-.9013	.1994
			committed athletes	-.1374	.26164	1.000	-.8339	.5592
			academically unengaged athletes	-.0763	.20760	1.000	-.6290	.4764
		academically unengaged athletes	fully engaged student-athletes	-.2746	.16915	.636	-.7250	.1758
			committed athletes	-.0610	.23310	1.000	-.6817	.5596
			academics-focused student-athletes	.0763	.20760	1.000	-.4764	.6290

psychological_well_being_MHC_SF	Bonferroni	fully engaged student-athletes	committed athletes	.2349	.21199	1.000	-.3295	.7993
			academics-focused student-athletes	.2597	.18863	1.000	-.2426	.7619
			academically unengaged athletes	.6819*	.15436	.000	.2709	1.0929
		committed athletes	fully engaged student-athletes	-.2349	.21199	1.000	-.7993	.3295
			academics-focused student-athletes	.0247	.23875	1.000	-.6109	.6604
			academically unengaged athletes	.4470	.21271	.221	-.1194	1.0133
		academics-focused student-athletes	fully engaged student-athletes	-.2597	.18863	1.000	-.7619	.2426
			committed athletes	-.0247	.23875	1.000	-.6604	.6109
			academically unengaged athletes	.4222	.18944	.161	-.0821	.9266
		academically unengaged athletes	fully engaged student-athletes	-.6819*	.15436	.000	-1.0929	-.2709
			committed athletes	-.4470	.21271	.221	-1.0133	.1194
			academics-focused student-athletes	-.4222	.18944	.161	-.9266	.0821

Based on observed means.

The error term is Mean Square(Error) = ,929.

*. The mean difference is significant at the .05 level.
